# Bis(2-amino-1,3-thia­zole-κ*N* ^3^)diazido­zinc

**DOI:** 10.1107/S1600536810053766

**Published:** 2011-01-08

**Authors:** Seung Wook Suh, Chong-Hyeak Kim, Inn Hoe Kim

**Affiliations:** a Department of Chemistry, Konyang University, Nonsan 320-711, Republic of Korea; b Center for Chemical Analysis, Korea Research Institute of Chemical Technology, PO Box 107, Yuseong, Daejeon 305-600, Republic of Korea

## Abstract

In the title complex, [Zn(N_3_)_2_(C_3_H_4_N_2_S)_2_], the Zn^II^ atom is tetra­hedrally coordinated by two terminal azide ligands and by the ring N atoms of two different 2-amino­thia­zole ligands. Intra­molecular N—H⋯N hydrogen bonds between the amino groups of both 2-amino­thia­zole ligands and the N atom of one of the azide ligands ensure that the heterocyclic rings are oriented in the same direction. Inter­molecular N—H⋯N hydrogen bonds link the mol­ecules into zigzag sheets in the *ac* plane.

## Related literature

For multi-dimensional supra­molecular complexes with organic–inorganic hybrids, see: Iwamoto (1996 ▶); Batten & Robson (1998 ▶); Braga *et al.* (1998 ▶). For the use of pseudo-halides in the construction of supra­molecular assemblies, see: Vrieze & Koten (1987 ▶); Cortes *et al.* (1997 ▶); Yun *et al.* (2004 ▶); Kim *et al.* (2008 ▶). For the coordination chemistry of imidazole and thia­zole derivatives, see: Costes *et al.* (1991 ▶); Balch *et al.* (1993 ▶); Suh *et al.* (2005 ▶, 2007 ▶, 2009 ▶); Kim & Kim (2010 ▶).
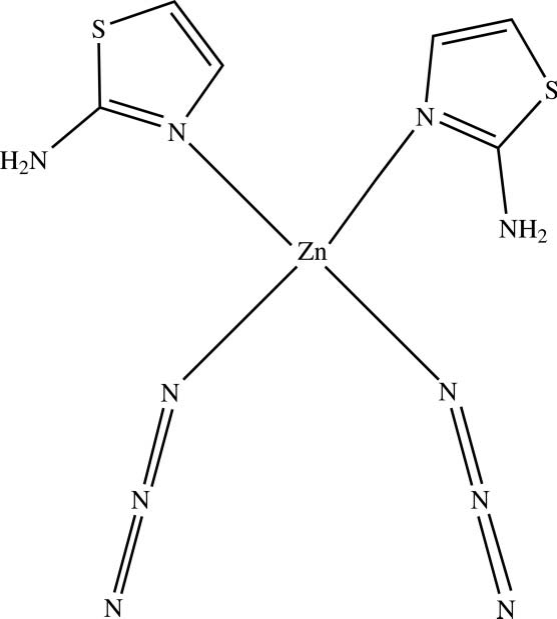


## Experimental

### 

#### Crystal data

[Zn(N_3_)_2_(C_3_H_4_N_2_S)_2_]
*M* *_r_* = 349.71
Triclinic, 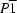

*a* = 8.096 (1) Å
*b* = 8.4004 (8) Å
*c* = 10.066 (1) Å
α = 96.489 (9)°
β = 100.66 (1)°
γ = 96.885 (9)°
*V* = 661.5 (1) Å^3^
*Z* = 2
Mo *K*α radiation
μ = 2.18 mm^−1^
*T* = 295 K
0.42 × 0.38 × 0.24 mm

#### Data collection

Bruker P4 diffractometer
Absorption correction: ψ scan (North *et al.*, 1968 ▶) *T*_min_ = 0.462, *T*_max_ = 0.623
3352 measured reflections
2747 independent reflections
2544 reflections with *I* > 2σ(*I*)
*R*_int_ = 0.013
3 standard reflections every 97 reflections  intensity decay: none

#### Refinement

*R*[*F* ^2^ > 2σ(*F* ^2^)] = 0.025
*wR*(*F* ^2^) = 0.068
*S* = 1.09
2747 reflections
173 parameters
H-atom parameters constrained
Δρ_max_ = 0.29 e Å^−3^
Δρ_min_ = −0.28 e Å^−3^

### 

Data collection: *XSCANS* (Bruker, 1996 ▶); cell refinement: *XSCANS*; data reduction: *SHELXTL* (Sheldrick, 2008 ▶); program(s) used to solve structure: *SHELXS97* (Sheldrick, 2008 ▶); program(s) used to refine structure: *SHELXL97* (Sheldrick, 2008 ▶); molecular graphics: *SHELXTL*; software used to prepare material for publication: *SHELXTL*.

## Supplementary Material

Crystal structure: contains datablocks global, I. DOI: 10.1107/S1600536810053766/pk2293sup1.cif

Structure factors: contains datablocks I. DOI: 10.1107/S1600536810053766/pk2293Isup2.hkl

Additional supplementary materials:  crystallographic information; 3D view; checkCIF report

## Figures and Tables

**Table 1:** Hydrogen-bond geometry (Å, °)

*D*—H⋯*A*	*D*—H	H⋯*A*	*D*⋯*A*	*D*—H⋯*A*
N16—H16*A*⋯N4	0.86	2.30	3.080 (3)	151
N16—H16*A*⋯N6^i^	0.86	2.57	3.033 (3)	115
N16—H16*B*⋯N3^ii^	0.86	2.34	3.102 (3)	148
N26—H26*A*⋯N4	0.86	2.24	3.005 (3)	148
N26—H26*B*⋯N3^iii^	0.86	2.28	3.071 (3)	153

Symmetry codes: (i) 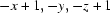; (ii) 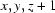; (iii) 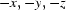.

**Fig. 1.**
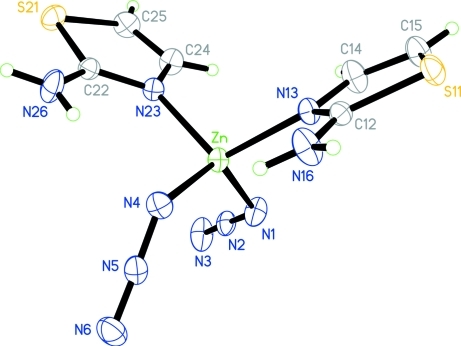
The molecular structure of the title complex with the atomic numbering and 30% probability displacement ellipsoids. H atoms are shown as small spheres of arbitary radius.

**Fig. 2.**
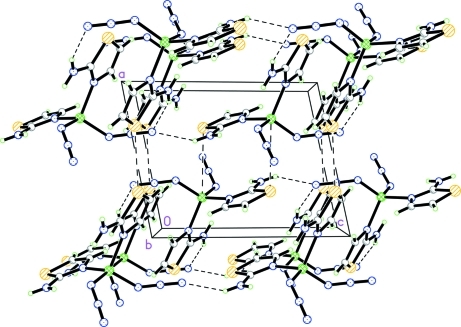
The crystal packing diagram of the title complex, viewed down the b axis showing the N—H···N(dashed lines) hydrogen bonds.

## supplementary crystallographic information

### Comment

Multi-dimensional supramolecular complexes with both organic and inorganic ligands have become of great interest recently (Iwamoto, 1996; Batten & Robson, 1998). They have been shown to have useful electronic, magnetic, optical, catalytic, *etc.* properties (Braga *et al.*, 1998). For designing novel 1-, 2- and 3-D frameworks, we (Kim *et al.*, 2008) and others (Cortes *et al.*, 1997; Yun *et al.*, 2004) have used the coordination properties of pseudohalide ions and complementary organic ligands. Pseudo-halide ions are known to build up 1-, 2- and 3-D structures by bridging metal centers (Vrieze & Koten, 1987). The of use of complementary organic ligands, such as aliphatic and aromatic amines is known to play an important role in stabilizing multi-dimensional structures. In particular, aromatic heterocycles such as imidazole and thiazole derivatives represent an important class of ligands in coordination chemistry (Balch *et al.*, 1993; Costes *et al.*, 1991). However, the frameworks of metal complexes with thiazole derivatives have been considerably less investigated. Our research is focused on the development of novel supramolecular structures utilizing the terminal and bridging properties of pseudo-halide ions, and the coordination behaviour of thiazole derivatives as complementary organic ligands (Suh *et al.*, 2005, 2007, 2009; Kim & Kim, 2010).  Herein, we present the synthesis and structure determination of the title complex, Zn(N_3_)_2_(C_3_H_4_N_2_S)_2_, with 2-aminothiazole as shown in Fig. 1. In the title complex, the Zn^II^ atom is tetrahedrally coordinated by two terminal azido ligands, and by the N atoms of two different 2-aminothiazole ligands.  Intramolecular N—H···N hydrogen bonds between the amino groups of both 2-aminothiazole ligands and the nitrogen atom of one of the azido ligands ensure that the heterocyclic rings are oriented in the same direction.  Intermolecular N—H···N hydrogen bonds form the molecules into zig-zag sheets in the *ac* plane (Fig. 2).

### Experimental

A water-methanolic (2:1) solution (60 ml) of sodium azide (9 mmol, 0.59 g) was added to a water-methanolic (2:1) solution (50 ml) of ZnSO_4_.7H_2_O (3 mmol, 0.87 g). To this mixture, a water-methanolic (2:1) solution (80 ml) of 2-aminobenzothiazole (10 mmol, 1.00 g) was introduced, with stirring for 1 h. The small amount of precipitates formed from the resulting solution were filtered off. The filtered solution was allowed to stand at room temperature. After a 1 week dark-yellow block crystals suitable for X-ray analysis were obtained. Elemental analysis found: C 20.73, H 2.25, N 40.22, S 18.50, Zn 18.70%; C_6_H_8_N_10_S_2_Zn requires: C 20.61, H 2.31, N 40.05, S 18.34, Zn 18.76%.

### Refinement

All H atoms were placed in calculated positions using a riding model, with C—H = 0.93 Å and *U*_iso_(H) = 1.2 *U*_eq_(C) for hetrocyclic H atoms and N—H = 0.86 Å and *U*_iso_(H) = 1.2 *U*_eq_(N) for amino H atom.

### Crystal data

**Table:**

[Zn(N_3_)_2_(C_3_H_4_N_2_S)_2_]	*Z* = 2
*M**_r_* = 349.71	*F*(000) = 352
Triclinic, *P*1	*D*_x_ = 1.756 Mg m^−^^3^*D*_m_ = 1.76 Mg m^−^^3^*D*_m_ measured by flotation method
Hall symbol: -P 1	Mo *K*α radiation, λ = 0.71073 Å
*a* = 8.096 (1) Å	Cell parameters from 39 reflections
*b* = 8.4004 (8) Å	θ = 4.7–14.6°
*c* = 10.066 (1) Å	µ = 2.18 mm^−^^1^
α = 96.489 (9)°	*T* = 295 K
β = 100.66 (1)°	Block, dark yellow
γ = 96.885 (9)°	0.42 × 0.38 × 0.24 mm
*V* = 661.5 (1) Å^3^	

### Data collection

**Table:**

Bruker P4 diffractometer	2544 reflections with *I* > 2σ(*I*)
Radiation source: fine-focus sealed tube	*R*_int_ = 0.013
graphite	θ_max_ = 26.5°, θ_min_ = 2.1°
2θ/ω scans	*h* = −1→10
Absorption correction: ψ scan (North *et al.*, 1968)	*k* = −10→10
*T*_min_ = 0.462, *T*_max_ = 0.623	*l* = −12→12
3352 measured reflections	3 standard reflections every 97 reflections
2747 independent reflections	intensity decay: none

### Refinement

**Table:**

Refinement on *F*^2^	Secondary atom site location: difference Fourier map
Least-squares matrix: full	Hydrogen site location: inferred from neighbouring sites
*R*[*F*^2^ > 2σ(*F*^2^)] = 0.025	H-atom parameters constrained
*wR*(*F*^2^) = 0.068	*w* = 1/[σ^2^(*F*_o_^2^) + (0.0243*P*)^2^ + 0.3124*P*] where *P* = (*F*_o_^2^ + 2*F*_c_^2^)/3
*S* = 1.09	(Δ/σ)_max_ < 0.001
2747 reflections	Δρ_max_ = 0.29 e Å^−^^3^
173 parameters	Δρ_min_ = −0.28 e Å^−^^3^
0 restraints	Extinction correction: *SHELXL97* (Sheldrick, 2008), Fc^*^=kFc[1+0.001xFc^2^λ^3^/sin(2θ)]^-1/4^
Primary atom site location: structure-invariant direct methods	Extinction coefficient: 0.0118 (11)

### Special details

**Table:**

Geometry. All e.s.d.'s (except the e.s.d. in the dihedral angle between two l.s. planes) are estimated using the full covariance matrix. The cell e.s.d.'s are taken into account individually in the estimation of e.s.d.'s in distances, angles and torsion angles; correlations between e.s.d.'s in cell parameters are only used when they are defined by crystal symmetry. An approximate (isotropic) treatment of cell e.s.d.'s is used for estimating e.s.d.'s involving l.s. planes.
Refinement. Refinement of *F*^2^ against ALL reflections. The weighted *R*-factor *wR* and goodness of fit *S* are based on *F*^2^, conventional *R*-factors *R* are based on *F*, with *F* set to zero for negative *F*^2^. The threshold expression of *F*^2^ > σ(*F*^2^) is used only for calculating *R*-factors(gt) *etc*. and is not relevant to the choice of reflections for refinement. *R*-factors based on *F*^2^ are statistically about twice as large as those based on *F*, and *R*- factors based on ALL data will be even larger.

### Fractional atomic coordinates and isotropic or equivalent isotropic displacement parameters (Å^2^)

**Table:**

	*x*	*y*	*z*	*U*_iso_*/*U*_eq_	
Zn	0.22961 (3)	0.28874 (3)	0.27010 (2)	0.03913 (10)	
N1	0.3512 (3)	0.4038 (3)	0.1481 (2)	0.0578 (5)	
N2	0.3327 (3)	0.3684 (2)	0.0289 (2)	0.0479 (4)	
N3	0.3184 (4)	0.3406 (3)	−0.0866 (2)	0.0758 (8)	
N4	0.3014 (3)	0.1005 (2)	0.3536 (2)	0.0496 (5)	
N5	0.4122 (3)	0.0255 (2)	0.32922 (19)	0.0475 (5)	
N6	0.5156 (3)	−0.0496 (3)	0.3098 (3)	0.0689 (6)	
S11	0.28221 (10)	0.65810 (8)	0.64496 (7)	0.05854 (18)	
C12	0.2849 (3)	0.4728 (3)	0.5514 (2)	0.0407 (4)	
N13	0.2265 (3)	0.4669 (2)	0.41986 (18)	0.0434 (4)	
C14	0.1766 (4)	0.6139 (3)	0.3920 (3)	0.0646 (7)	
H14A	0.1319	0.6314	0.3038	0.077*	
C15	0.1962 (4)	0.7276 (3)	0.4978 (3)	0.0655 (7)	
H15	0.1674	0.8310	0.4931	0.079*	
N16	0.3412 (3)	0.3506 (3)	0.6102 (2)	0.0649 (6)	
H16A	0.3410	0.2596	0.5614	0.078*	
H16B	0.3779	0.3623	0.6970	0.078*	
S21	−0.28571 (8)	0.04332 (9)	0.02647 (7)	0.05992 (18)	
C22	−0.0995 (3)	0.0694 (3)	0.1466 (2)	0.0424 (5)	
N23	−0.0062 (2)	0.2108 (2)	0.15549 (18)	0.0408 (4)	
C24	−0.0868 (3)	0.3032 (3)	0.0654 (3)	0.0561 (6)	
H24A	−0.0395	0.4079	0.0589	0.067*	
C25	−0.2352 (4)	0.2348 (3)	−0.0113 (3)	0.0616 (7)	
H25	−0.3018	0.2835	−0.0760	0.074*	
N26	−0.0579 (3)	−0.0446 (3)	0.2244 (3)	0.0672 (7)	
H26A	0.0349	−0.0273	0.2847	0.081*	
H26B	−0.1241	−0.1351	0.2141	0.081*	

### Atomic displacement parameters (Å^2^)

**Table:**

	*U*^11^	*U*^22^	*U*^33^	*U*^12^	*U*^13^	*U*^23^
Zn	0.04775 (16)	0.03781 (14)	0.03000 (14)	0.00320 (10)	0.00434 (10)	0.00587 (9)
N1	0.0701 (14)	0.0594 (12)	0.0389 (10)	−0.0144 (11)	0.0145 (10)	0.0049 (9)
N2	0.0579 (12)	0.0396 (9)	0.0457 (11)	−0.0058 (8)	0.0160 (9)	0.0081 (8)
N3	0.117 (2)	0.0644 (14)	0.0413 (12)	−0.0165 (14)	0.0270 (13)	0.0021 (10)
N4	0.0558 (12)	0.0490 (11)	0.0446 (10)	0.0134 (9)	0.0044 (9)	0.0130 (8)
N5	0.0559 (12)	0.0406 (10)	0.0401 (10)	0.0058 (9)	−0.0050 (8)	0.0070 (8)
N6	0.0728 (16)	0.0597 (14)	0.0763 (16)	0.0273 (12)	0.0086 (13)	0.0100 (12)
S11	0.0711 (4)	0.0552 (3)	0.0448 (3)	0.0152 (3)	0.0066 (3)	−0.0102 (3)
C12	0.0433 (11)	0.0447 (11)	0.0333 (10)	0.0047 (9)	0.0090 (8)	0.0020 (8)
N13	0.0566 (11)	0.0393 (9)	0.0330 (8)	0.0074 (8)	0.0056 (8)	0.0050 (7)
C14	0.096 (2)	0.0496 (14)	0.0472 (13)	0.0235 (14)	0.0030 (14)	0.0101 (11)
C15	0.085 (2)	0.0483 (14)	0.0629 (16)	0.0222 (14)	0.0088 (15)	0.0033 (12)
N16	0.1032 (19)	0.0590 (13)	0.0313 (10)	0.0276 (13)	0.0015 (11)	0.0047 (9)
S21	0.0440 (3)	0.0662 (4)	0.0607 (4)	0.0020 (3)	−0.0054 (3)	0.0040 (3)
C22	0.0405 (11)	0.0445 (11)	0.0406 (11)	0.0059 (9)	0.0053 (9)	0.0037 (9)
N23	0.0439 (10)	0.0395 (9)	0.0381 (9)	0.0061 (7)	0.0035 (7)	0.0088 (7)
C24	0.0614 (15)	0.0534 (14)	0.0525 (14)	0.0089 (12)	0.0011 (12)	0.0201 (11)
C25	0.0622 (16)	0.0697 (17)	0.0516 (14)	0.0199 (13)	−0.0030 (12)	0.0163 (12)
N26	0.0603 (14)	0.0470 (11)	0.0853 (17)	−0.0080 (10)	−0.0084 (12)	0.0262 (11)

### Geometric parameters (Å, °)

**Table:**

Zn—N4	1.9711 (19)	C14—H14A	0.9300
Zn—N1	1.974 (2)	C15—H15	0.9300
Zn—N13	2.0066 (18)	N16—H16A	0.8600
Zn—N23	2.0292 (18)	N16—H16B	0.8600
N1—N2	1.181 (3)	S21—C25	1.714 (3)
N2—N3	1.140 (3)	S21—C22	1.723 (2)
N4—N5	1.202 (3)	C22—N23	1.313 (3)
N5—N6	1.138 (3)	C22—N26	1.339 (3)
S11—C15	1.712 (3)	N23—C24	1.384 (3)
S11—C12	1.730 (2)	C24—C25	1.326 (4)
C12—N13	1.314 (3)	C24—H24A	0.9300
C12—N16	1.327 (3)	C25—H25	0.9300
N13—C14	1.386 (3)	N26—H26A	0.8600
C14—C15	1.320 (4)	N26—H26B	0.8600
			
N4—Zn—N1	124.47 (10)	C14—C15—H15	124.8
N4—Zn—N13	108.49 (8)	S11—C15—H15	124.8
N1—Zn—N13	102.24 (8)	C12—N16—H16A	120.0
N4—Zn—N23	105.71 (8)	C12—N16—H16B	120.0
N1—Zn—N23	104.23 (8)	H16A—N16—H16B	120.0
N13—Zn—N23	111.56 (8)	C25—S21—C22	89.76 (12)
N2—N1—Zn	125.88 (17)	N23—C22—N26	124.1 (2)
N3—N2—N1	177.1 (2)	N23—C22—S21	113.83 (17)
N5—N4—Zn	127.06 (17)	N26—C22—S21	122.09 (18)
N6—N5—N4	177.2 (3)	C22—N23—C24	110.0 (2)
C15—S11—C12	89.52 (12)	C22—N23—Zn	127.90 (15)
N13—C12—N16	124.6 (2)	C24—N23—Zn	121.96 (16)
N13—C12—S11	113.59 (17)	C25—C24—N23	116.5 (2)
N16—C12—S11	121.83 (17)	C25—C24—H24A	121.7
C12—N13—C14	110.16 (19)	N23—C24—H24A	121.7
C12—N13—Zn	127.78 (16)	C24—C25—S21	109.9 (2)
C14—N13—Zn	121.67 (16)	C24—C25—H25	125.1
C15—C14—N13	116.3 (2)	S21—C25—H25	125.1
C15—C14—H14A	121.9	C22—N26—H26A	120.0
N13—C14—H14A	121.9	C22—N26—H26B	120.0
C14—C15—S11	110.5 (2)	H26A—N26—H26B	120.0
			
N4—Zn—N1—N2	86.1 (3)	C12—N13—C14—C15	0.0 (4)
N13—Zn—N1—N2	−151.0 (2)	Zn—N13—C14—C15	−173.3 (2)
N23—Zn—N1—N2	−34.7 (3)	N13—C14—C15—S11	0.3 (4)
Zn—N1—N2—N3	164 (6)	C12—S11—C15—C14	−0.4 (3)
N1—Zn—N4—N5	−8.6 (2)	C25—S21—C22—N23	−0.86 (19)
N13—Zn—N4—N5	−128.7 (2)	C25—S21—C22—N26	178.0 (2)
N23—Zn—N4—N5	111.6 (2)	N26—C22—N23—C24	−177.6 (2)
Zn—N4—N5—N6	−177 (100)	S21—C22—N23—C24	1.2 (3)
C15—S11—C12—N13	0.5 (2)	N26—C22—N23—Zn	6.5 (3)
C15—S11—C12—N16	−179.4 (2)	S21—C22—N23—Zn	−174.66 (10)
N16—C12—N13—C14	179.5 (3)	N4—Zn—N23—C22	5.3 (2)
S11—C12—N13—C14	−0.4 (3)	N1—Zn—N23—C22	138.0 (2)
N16—C12—N13—Zn	−7.8 (4)	N13—Zn—N23—C22	−112.44 (19)
S11—C12—N13—Zn	172.39 (11)	N4—Zn—N23—C24	−170.06 (19)
N4—Zn—N13—C12	9.6 (2)	N1—Zn—N23—C24	−37.4 (2)
N1—Zn—N13—C12	−123.5 (2)	N13—Zn—N23—C24	72.2 (2)
N23—Zn—N13—C12	125.6 (2)	C22—N23—C24—C25	−1.0 (3)
N4—Zn—N13—C14	−178.4 (2)	Zn—N23—C24—C25	175.2 (2)
N1—Zn—N13—C14	48.5 (2)	N23—C24—C25—S21	0.3 (3)
N23—Zn—N13—C14	−62.3 (2)	C22—S21—C25—C24	0.3 (2)

### Hydrogen-bond geometry (Å, °)

**Table:**

*D*—H···*A*	*D*—H	H···*A*	*D*···*A*	*D*—H···*A*
N16—H16A···N4	0.86	2.30	3.080 (3)	151
N16—H16A···N6^i^	0.86	2.57	3.033 (3)	115
N16—H16B···N3^ii^	0.86	2.34	3.102 (3)	148
N26—H26A···N4	0.86	2.24	3.005 (3)	148
N26—H26B···N3^iii^	0.86	2.28	3.071 (3)	153

Symmetry codes: (i) −*x*+1, −*y*, −*z*+1; (ii) *x*, *y*, *z*+1; (iii) −*x*, −*y*, −*z*.

## References

[bb1] Balch, A. L., Noll, B. C. & Safari, N. (1993). *Inorg. Chem.* **32**, 2901–2905.

[bb2] Batten, S. R. & Robson, R. (1998). *Angew. Chem. Int. Ed.* **37**, 1460–1494. 10.1002/(SICI)1521-3773(19980619)37:11<1460::AID-ANIE1460>3.0.CO;2-Z 29710936

[bb3] Braga, D., Grepioni, F. & Desiraju, G. R. (1998). *Chem. Rev.* **98**, 1375–1406. 10.1021/cr960091b 11848937

[bb4] Bruker (1996). *XSCANS* Bruker AXS Inc., Madison, Wisconsin, USA.

[bb5] Cortes, R., Urtiaga, M. K., Lezama, L., Pizarro, J. L., Arriortua, M. I. & Rojo, T. (1997). *Inorg. Chem.* **36**, 5016–5021.

[bb6] Costes, J. P., Dahan, F. & Laurent, J. P. (1991). *Inorg. Chem.* **30**, 1887–1892.

[bb7] Iwamoto, T. (1996). *Comprehensive Supramolecular Chemistry*, Vol. 6, pp. 643–690. Oxford: Pergamon Press.

[bb8] Kim, C.-H. & Kim, I. H. (2010). *Acta Cryst.* E**66**, m13.

[bb9] Kim, C. H., Moon, H. S. & Lee, S. G. (2008). *Anal. Sci. Technol.* **21**, 562–568.

[bb10] North, A. C. T., Phillips, D. C. & Mathews, F. S. (1968). *Acta Cryst.* A**24**, 351–359.

[bb11] Sheldrick, G. M. (2008). *Acta Cryst.* A**64**, 112–122. 10.1107/S0108767307043930 18156677

[bb12] Suh, S. W., Kim, I. H. & Kim, C. H. (2005). *Anal. Sci. Technol.* **18**, 386–390.

[bb13] Suh, S. W., Kim, C.-H. & Kim, I. H. (2007). *Acta Cryst.* E**63**, m2177.

[bb14] Suh, S. W., Kim, C.-H. & Kim, I. H. (2009). *Acta Cryst.* E**65**, m1054. 10.1107/S1600536809030931 PMC2969946 21577414

[bb15] Vrieze, K. & Koten, G. V. (1987). *Comprehensive Coordination Chemistry*, Vol. 2, pp. 225–244. Oxford: Pergamon Press.

[bb16] Yun, S. S., Moon, H. S., Kim, C. H. & Lee, S. G. (2004). *J. Coord. Chem.* **57**, 321–327.

